# Supplementary material for: Collaborative intelligence in AI: Evaluating the performance of a council of AIs on the USMLE
Source: PLOS Digit Health. 2025 Oct 9;4(10):e0000787. doi: 10.1371/journal.pdig.0000787 (PMC12510544; doi:10.1371/journal.pdig.0000787)
Supplement: S2 File — (ZIP) [file pdig.0000787.s002.zip › S2 Data Raw data files containing unprocessed question inputs and LLM outputs including transcripts of deliberations/STEP3_Question-128-1_Type-MC-NJ.docx]

**Facilitator** **[Round 1]:** A 34-year-old woman, gravida 2, para 1, comes to the office at 26 weeks' gestation to establish prenatal care after obtaining health insurance coverage. She has gained 14 kg (31 lb) during this pregnancy. Her last pregnancy 4 years ago resulted in delivery of a 4564-g (10-lb 1-oz) male newborn. Today, the patient is 162 cm (5 ft 4 in) tall and weighs 99 kg (218 lb). Vital signs are normal. Physical examination discloses a fundal height of 30 cm. Fetal heart rate is 146/min. Prenatal laboratory studies and ultrasonography are ordered. This patient is most at risk for which of the following? (A) Gestational diabetes (B) Multiple gestation (C) Placenta previa (D) Polyhydramnios (E) Preeclampsia

**AI_B:** RESTATING THE QUESTION: The situation involves a 34-year-old woman, pregnant for the second time, now in her 26th week of gestation. She gained significant weight during this pregnancy, and her previous child was large (macrosomia). All physical parameters are normal at present, including the fetal heart rate. We need to identify the complication she's most likely to experience during this pregnancy among gestational diabetes, multiple gestation, placenta previa, polyhydramnios, and preeclampsia. PLANNING: The assumptions involved here are the risk factors and associative pathologies for each of these conditions. Gestational diabetes might be associated with overweight or obesity and a history of delivering a large baby (macrosomia). Multiple gestation would usually present with a higher fundal height than expected for the gestational age. Placenta previa might have symptoms like painless vaginal bleeding. Polyhydramnios could present with a larger-than-normal uterus. Preeclampsia would often include symptoms such as hypertension and proteinuria. The primary underlying assumption here is that previous obstetric history, current pregnancy weight, and her current health condition contribute to the risk of these complications. CLINICAL REASONING: Given her weight gain during pregnancy, her previous history of delivering a macrosomic baby (which can be a consequence of undiagnosed gestational diabetes in the previous pregnancy), and the absence of symptoms indicative of placenta previa, preeclampsia, multiple gestation, and polyhydramnios, gestational diabetes becomes the primary consideration. We know that obesity is a strong risk factor for the development of gestational diabetes, and given her Body Mass Index (BMI), this woman could be considered obese. Importantly, the incidences of gestational diabetes rises with increasing maternal age, and our patient is 34 years old. CONCLUDING: The woman's characteristics—significant weight gain in pregnancy, a previous delivery of a large baby, and obesity—point toward an increased risk of gestational diabetes. Other options such as preeclampsia, placenta previa, multiple gestation, and polyhydramnios do not align with the given clinical indications. RESPONSE: The patient is most at risk for gestational diabetes.

**AI_A:** RESTATING THE QUESTION: This is about a pregnant woman who is in her second pregnancy and currently at 26 weeks of gestation. She has significantly gained weight during her pregnancy and her prenatal checkup reveals a fundal height of 30 cm and a normal fetal heart rate. With the information provided, we are supposed to identify the most probable risk she's prone to among the options provided: gestational diabetes, multiple gestation, placenta previa, polyhydramnios or preeclampsia. PLANNING: The assumptions made here include: her previous delivery of a big baby, excessive weight gain, the fundal height and the normal fetal heart rate. Let's generate alternate assumptions by questioning each one: Could her delivery of a large baby in her past pregnancy put her at risk for gestational diabetes this time? Could her amount of weight gain be indicative of multiple gestation? Or maybe the excessive weight gain could be associated with conditions like polyhydramnios or preeclampsia? The fundal height seems larger than expected for her gestation period, so could this suggest conditions like polyhydramnios or multiple gestation, or is it simply due to obesity? Lastly, her normal fetal heart rate, does it rule out any of these conditions? Based on clinical significance and prevalence, the assumption of gestational diabetes due to her past delivery of a large baby seems a crucial one to evaluate. CLINICAL REASONING: A history of delivering a big baby (macrosomia) could possibly indicate that the mother may have had untreated gestational diabetes during that pregnancy. Uncontrolled gestational diabetes leads to excessive fetal growth due to high sugar levels crossing the placenta. This could place the mother at an increased risk for gestational diabetes in this current pregnancy. Furthermore, the patient’s excessive weight gain in her current pregnancy may also be associated with gestational diabetes; increased body weight can increase insulin resistance, which is a key component of gestational diabetes. CONCLUDING: The reasoning process leads to the conclusion that due to her history of a large baby in her previous pregnancy, and her above-average weight gain in her current pregnancy, the most significant risk for this patient could be gestational diabetes. RESPONSE: The patient is most at risk for option (A) Gestational diabetes.

**AI_D:** RESTATING THE QUESTION: The patient, a 34-year-old woman who is at her 26th week of gestation, has gained significant weight during this pregnancy, and has had a previous history of delivering a heavy newborn. She appears overall physically healthy at this stage of her pregnancy, with her vitals and physical examination results being within normal parameters. The query seeks to know what potential risk she may be prone to given these considerations: gestational diabetes, multiple gestation, placenta previa, polyhydramnios, or preeclampsia. PLANNING: Understanding the terms and parameters given, each condition carries a specific set of risk factors. Gestational diabetes is a condition where a woman without diabetes develops high blood sugar levels during pregnancy, typically linked to obesity, family history of diabetes, and previous history of gestational diabetes or a macrosomic baby (like the patient's first child). Multiple gestation means more than one fetus within the uterus and would often correspond to a higher fundal height in proportion to the gestational age. Placenta previa involves the placenta covering the cervix that usually presents with painless vaginal bleeding, while polyhydramnios involves an excessive amount of amniotic fluid. Lastly, preeclampsia is a complication of pregnancy characterized by high blood pressure and often a significant amount of protein in the urine. CLINICAL REASONING: The patient's information reveals a previous history of delivering a heavy baby and significant weight gain during this pregnancy (14 kg). Such factors are known to be associated with gestational diabetes. The fundal height of 30 cm is normal for the gestational age (26 weeks) and aligns with a single fetus pregnancy, excluding the probability of a multiple gestation. The absence of any bleeding or hypertension symptoms excludes placenta previa and preeclampsia. Nothing suggests an abnormal amount of amniotic fluid, which would indicate polyhydramnios - such diagnosis would generally require an ultrasound. CONCLUDING: We should focus on the conditions that relate to the signs presented by the patient. Given the significant weight gain during her current pregnancy and a previous history of delivering a relatively heavy baby, which are known risk factors, this patient is most at risk for gestational diabetes. RESPONSE: This patient is most at risk for (A) Gestational Diabetes.

**AI_E:** (1) RESTATING THE QUESTION: The query involves determining the highest risk medical condition for a 34-year-old pregnant woman who is in her second pregnancy and into her 26th week of gestation. This woman presents with a remarkable weight gain of 14 kg during this pregnancy. Her vital signs and fetal heart rate are within the normal range. Her first pregnancy resulted in the delivery of a newborn with a notably heavier weight of 4564 g. (2) PLANNING: The critical assumptions to analyze would be the patient's significant weight gain during her current pregnancy along with the history of delivering a larger-than-average newborn, which represents macrosomia in her previous pregnancy. A strong argument for gestational diabetes (GD) could be made based on the patient's high weight gain in this pregnancy and her history of a prior macrosomic baby, both of which are risk factors for GD. GD results from impaired glucose tolerance during pregnancy leading to high blood glucose levels, which in turn can cause excessive growth and high birth weight (macrosomia) in infants. The presence of a poorer obstetric history like multiple gestation, placenta previa, polyhydramnios, and preeclampsia too could lead to certain maternal and fetal complications, but these are not as strongly connected to the symptoms presented by the patient. (3) CLINICAL REASONING: Currently, she exhibits a fundal height of 30 cm at 26 weeks' gestation, which is on the higher side and could indicate an overly large fetus or excess amniotic fluid (polyhydramnios). But her excessive weight gain and history of a macrosomic newborn are most often associated with GD. In GD, the extra glucose in the mother’s bloodstream crosses the placenta leading to overgrowth of the fetus, also known as macrosomia. If untreated, GD can lead to complications for both the mother and fetus, including a larger birth weight for infants that can make delivery more challenging. (4) CONCLUDING: Although the patient's exaggerated fundal height suggests an increase in fetal size or fluid, the most telling signs - significant weight gain and the birth of a macrosomic baby in her previous pregnancy - point to the risk of gestational diabetes as the top concern. (5) RESPONSE: So, the patient is most at risk for (A) Gestational Diabetes. However, it's essential to await the results of her prenatal laboratory studies and ultrasonography, which could provide more concrete information.

**AI_C:** Restating the question: The question asks about the main health risk for a 34-year-old pregnant woman who is currently on her second pregnancy at 26 weeks' gestation, with a significant weight gain of 14 kg (31 lb) during this current pregnancy. This patient had a history of having a large newborn during her last pregnancy 4 years ago. On her physical examination, they recorded a fundal height of 30 cm and a fetal heart rate of 146/min. Other tests including prenatal laboratory studies and ultrasonography are still pending. Planning: Given the patient's clinical details, it's important to ponder these assumptions: 1) the lady's significant weight gain during this pregnancy, 2) her history of delivering a large baby previously, and 3) the recorded fundal height and fetal heart rate. Possible health risks associated with these assumptions could be gestational diabetes, multiple gestation, placenta previa, polyhydramnios, or preeclampsia. However, these conditions carry different levels of probability and it's vital to clinically prioritize these assumptions based on the patient's signs and history. Clinical Reasoning: Analyzing our patient, her significant weight gain during this pregnancy, coupled with her history of delivering a large baby during her previous pregnancy, significantly increases the probability of her developing gestational diabetes. Gestational diabetes is a condition where there's higher-than-normal blood glucose levels during pregnancy. It's known to increase the risk of macrosomia, which is evident from the patient's history of having a large baby. Gestational diabetes can also contribute to a larger than normal fundal height, as it might be seen in our patient, due to the large size of the fetus. Furthermore, while conditions like multiple gestation, placenta previa, polyhydramnios, and preeclampsia are also possible within the context of pregnancy, they are less likely. For instance, there is no mention of an unusually large uterus for gestational age that would suggest multiple gestation or polyhydramnios. There are also no symptoms, such as vaginal bleeding, which could point towards placenta previa. Also, the normal vital signs would rule out preeclampsia, which usually features high blood pressure. In terms of the weight gain, this could be due to the pregnancy itself or could indicate gestational diabetes, as this condition can cause excessive weight gain. Looking at these different variables, it seems most likely that the greatest risk to the patient is gestational diabetes. Concluding: After considering the signs and history of the patient, the risk of gestational diabetes appears to be clinically prioritized over the other conditions listed. Response: Therefore, the patient is most at risk for (A) Gestational diabetes.

**Facilitator** The consensus statement synthesizes the responses of AI_A, AI_B, AI_C, AI_D, and AI_E as follows: The question pertains to a 34-year-old woman who is in her second pregnancy at 26 weeks' gestation. She has gained significant weight during this pregnancy and has a history of delivering a large baby in her previous pregnancy. Her physical examination reveals a fundal height of 30 cm and a normal fetal heart rate. The task is to identify the most probable risk she's prone to among the options provided: gestational diabetes, multiple gestation, placenta previa, polyhydramnios, or preeclampsia. The assumptions made here include her previous delivery of a big baby, excessive weight gain, the fundal height, and the normal fetal heart rate. A history of delivering a big baby (macrosomia) could possibly indicate that the mother may have had untreated gestational diabetes during that pregnancy. Uncontrolled gestational diabetes leads to excessive fetal growth due to high sugar levels crossing the placenta. This could place the mother at an increased risk for gestational diabetes in this current pregnancy. Furthermore, the patient’s excessive weight gain in her current pregnancy may also be associated with gestational diabetes; increased body weight can increase insulin resistance, which is a key component of gestational diabetes. Given her weight gain during pregnancy, her previous history of delivering a macrosomic baby (which can be a consequence of undiagnosed gestational diabetes in the previous pregnancy), and the absence of symptoms indicative of placenta previa, preeclampsia, multiple gestation, and polyhydramnios, gestational diabetes becomes the primary consideration. We know that obesity is a strong risk factor for the development of gestational diabetes, and given her Body Mass Index (BMI), this woman could be considered obese. Importantly, the incidences of gestational diabetes rises with increasing maternal age, and our patient is 34 years old. The woman's characteristics—significant weight gain in pregnancy, a previous delivery of a large baby, and obesity—point toward an increased risk of gestational diabetes. Other options such as preeclampsia, placenta previa, multiple gestation, and polyhydramnios do not align with the given clinical indications. Therefore, the patient is most at risk for (A) Gestational diabetes. However, it's essential to await the results of her prenatal laboratory studies and ultrasonography, which could provide more concrete information.
